# Endobronchial valves for emphysema and persistent air-leak: 10-year experience in an Asian country

**DOI:** 10.1186/s12890-024-02982-2

**Published:** 2024-04-03

**Authors:** Jin-Young Huh, Byeong-Ho Jeong, Ho il Yoon, Hojoong Kim, Young-Jae Cho, Changhwan Kim, Seung Jun Lee, Hwan hee Kim, Seung Won Ra, Ye Jin Lee, Beong Ki Kim, Sung Kyoung Kim, Ki Hyun Seo, Sei Won Lee

**Affiliations:** 1 Division of Pulmonary, Allergy and Critical Care Medicine, Department of Internal Medicine, Chung-Ang University Gwangmyeong Hospital, Gwangmyeong, South Korea; 2 Division of Pulmonary and Critical Care Medicine, Department of Medicine, Samsung Medical Center, Sungkyunkwan University School of Medicine, Seoul, South Korea; 3 Division of Pulmonary and Critical Care Medicine, Department of Internal Medicine, Seoul National University Bundang Hospital, Seongnam, South Korea; 4 Department of Internal Medicine, Jeju National University Hospital, Jeju National University School of Medicine, Jeju, South Korea; 5 Department of Internal Medicine, Hallym University Dongtan Sacred Heart Hospital, Hwaseong-si, Gyeonggi-do South Korea; 6 Department of Internal Medicine, Gyeongsang National University Hospital, Gyeongsang National University College of Medicine, Jinju, South Korea; 7 Division of Pulmonary, Critical Care and Sleep Medicine, Department of Internal Medicine, Eunpyeong St. Mary’s Hospital, College of Medicine, The Catholic University of Korea, Seoul, South Korea; 8 Department of Internal Medicine, Ulsan University Hospital, University of Ulsan College of Medicine, Ulsan, South Korea; 9 Division of Pulmonology, Allergy and Critical Care Medicine, Department of Internal Medicine, Korea University Ansan Hospital, Ansan, South Korea; 10 Department of Internal Medicine, St. Vincent’s Hospital, College of Medicine, The Catholic University of Korea, Suwon, South Korea; 11 Division of Pulmonary and Critical Care Medicine, Department of Medicine, Soonchunhyang University Cheonan Hospital, Cheonan, South Korea; 12 Department of Pulmonary and Critical Care Medicine, and Clinical Research Center for Chronic Obstructive Airway Diseases, Asan Medical Center, University of Ulsan College of Medicine, 88, Olympic-Ro 43-Gil, Songpa-Gu, Seoul, 05505 Republic of Korea

**Keywords:** Chronic obstructive pulmonary disease, Emphysema, Pneumothorax, Bronchoscopies

## Abstract

**Background:**

Endobronchial valve (EBV) therapy, a validated method for bronchoscopic lung volume reduction (BLVR) in severe emphysema, has been explored for persistent air-leak (PAL) management. However, its effectiveness and safety in the Asian population require further real-world evaluation. In this study, we assessed the outcomes of treatment with EBV within this demographic.

**Methods:**

We conducted a retrospective analysis of medical records from 11 Korean centers. For the emphysema cohort, inclusion criteria were patients diagnosed with emphysema who underwent bronchoscopy intended for BLVR. We assessed these patients for clinical outcomes of chronic obstructive pulmonary disease. All patients with PAL who underwent treatment with EBV were included. We identified the underlying causes of PAL and evaluated clinical outcomes after the procedure.

**Results:**

The severe emphysema cohort comprised 192 patients with an average age of 70.3 years, and 95.8% of them were men. Ultimately, 137 underwent treatment with EBV. Three months after the procedure, the BLVR group demonstrated a significant improvement in forced expiratory volume in 1 s (+160 mL vs. +30 mL; *P* = 0.009). Radiographic evidence of lung volume reduction 6 months after BLVR was significantly associated with improved survival (adjusted hazard ratio 0.020; 95% confidence interval 0.038–0.650; *P* = 0.010). Although pneumothorax was more common in the BLVR group (18.9% vs. 3.8%; *P* = 0.018), death was higher in the no-BLVR group (38.5% vs. 54.5%, *P* = 0.001), whereas other adverse events were comparable between the groups. Within the subset of 18 patients with PAL, the predominant causes of air-leak included spontaneous secondary pneumothorax (44.0%), parapneumonic effusion/empyema (22.2%), and post-lung resection surgery (16.7%). Following the treatment, the majority (77.8%) successfully had their chest tubes removed. Post-procedural complications were minimal, with two incidences of hemoptysis and one of empyema, all of which were effectively managed.

**Conclusions:**

Treatment with EBV provides substantial clinical benefits in the management of emphysema and PAL in the Asian population, suggesting a favorable outcome for this therapeutic approach.

**Supplementary Information:**

The online version contains supplementary material available at 10.1186/s12890-024-02982-2.

## Background

Bronchoscopic lung volume reduction (BLVR) was introduced in 2010 with the publication on the endobronchial valve (EBV) treatment for Emphysema Palliation Trial (VENT) [1]. The EBV is a minimally invasive bronchoscopic device designed for lung volume reduction in patients with severe emphysema. Its primary characteristics include the ability to redirect airflow away from hyperinflated areas of the lung, promoting improved lung function and systemic relief. Although adverse events include pneumothorax, acute exacerbation of chronic obstructive pulmonary disease (COPD), and hemoptysis, the incidences are relatively low at less than 8%. The procedure improved lung function, exercise capacity, and respiratory symptoms [1]. After the VENT trial, the absence of interlobar collateral ventilation (CV) was identified as an important predictor of response to BLVR [2]. Subsequent studies among selected patients without collateral ventilation showed consistent improvements in clinical parameters and acceptable safety profiles [3–5].

In 2018, the Zephyr endobronchial valve was approved by the United States Food and Drug Administration [6]. Since then, EBV has become a treatment option for advanced emphysema recommended by GOLD [7] and NICE [8].

In Asian countries, the clinical application of EBV has been relatively scarcely reported. The first reports of EBV in Asia, published in 2015 [9, 10], also showed improvements in lung function, exercise capacity, respiratory symptoms, and quality of life. Meanwhile, these studies also reported a higher complication rate of pneumothorax than that reported in studies conducted involving Western populations [1, 2, 11]. The possible explanations include an older target population, lower body mass index (BMI), and poorer exercise capacity in the Asian target population. Despite lower forced expiratory volume in 1 s (FEV_1_), hyperinflation, represented by residual volume, was less severe. The findings suggest some discrepancies in patient characteristics among ethnic groups, and thoracic cage morphology may be one of them [12].

Furthermore, there are not enough long-term studies on BLVR including the Asian population. In this study, we collected clinical data of the entire population considered for treatment with EBV in Korea to confirm its long-term efficacy and safety in Korea.

## Methods

### Study design and study population

This retrospective multicenter cohort study included patients from Korea between July 2012 and July 2021. A total of 13 institutions had experience with EBV during the study period, and 11 of them provided the data. The number of omitted patients was less than 15 based on the market survey.

The inclusion criterion was bronchoscopic assessment for treatment with EBV. The two distinct indications for EBV insertion; i) bronchoscopic lung volume reduction (BLVR) in patients with emphysema and ii) persistent air-leak (PAL) in patients with a chest tube, were considered for the study. Patients were selected at the attending physician’s discretion.

For BLVR candidates, the presence of collateral ventilation (CV) was a primary exclusion criterion due to its negative impact on the treatment outcomes. Most institutes included patients with a minimum Modified Medical Research Council (mMRC) dyspnea scale score of 2; however, one institute included patients with an mMRC score of 1, justifying their inclusion based on their severe airflow limitation. CV status was assessed physiologically with the Chartis^®^ Pulmonary Assessment System (PulmonX Corporation., Redwood City, CA, USA) and radiographically with computed tomography (CT)-fissure analysis. The Chartis system facilitated isolation of a target lobe using a balloon catheter, followed by measurement of airflow and pressure within the lobe. A key criterion for contraindicating BLVR was the detection of significant airflow in the lobe despite balloon occlusion, indicating substantial ventilation. Furthermore, BLVR was contraindicated if the fissure lengths were not clearly defined for 5–10% of their total length. Those who underwent bronchoscopy but did not receive EBV due to CV were classified into the no-BLVR group. All patients with persistent air-leak received treatment with EBV. The valves used in this study were Zephyr one-way EBV (PulmonX Corporation, Redwood City, CA, USA).

For the management of PAL, the treatment approach was uniformly inclusive across all centers. The assessment of collateral ventilation varied among the institutes; three utilized the Chartis system whereas the other three did not.

The present study was approved by the Institutional Review Board (IRB) of each institution. Due to the retrospective nature of the study, the need for informed consent was waived by the IRB of Asan Medical Center. The IRB protocol numbers are documented in the additional file (Additional file 1). The investigation was performed in accordance with the Declaration of Helsinki.

### Data collection

The clinical and survival data were retrospectively collected from medical records. Patients were followed from the date of bronchoscopic assessment until death or the last date of follow-up. Collected data included demographics, comorbidities, treatment for the lung disease, pulmonary function test (PFT) results, radiographic findings, chronic obstructive pulmonary disease (COPD) assessment test results, mMRC Dyspnoea Scale score, 6-min walk distance, procedure-related details, clinical outcomes, and adverse events following the procedure. The adverse events included pneumothorax, hemoptysis, empyema, acute exacerbation of COPD, and mortality.

### Effects after EBV insertion

Among patients requiring BLVR, all-cause mortality and incidents of acute exacerbation of COPD were evaluated. The events were compared between patients who received EBV and those who did not. Changes in FEV_1_ and radiographic findings before and after the index date were also compared. Changes in FEV_1_ were assessed by measuring differences in FEV1 before and after the index date. Lung volume reduction of the target lobe was assessed by the physicians at each participating study site. It was defined by a reduction of more than 50% in lung volume assessed through chest radiographs or chest CT scans obtained 6 months after the procedure. To ensure consistency and objectivity in these assessments, any cases of disagreement or ambiguity were resolved through a consensus decision made by JYH and SWL. Subgroup analyses of mortality and acute exacerbation were performed to determine the effects of response to BLVR. For patients with PAL treated off-label with EBV, we collected information on the air-leak etiology, specifics of the EBV procedure, the timing of chest tube removal, and any subsequent adverse events.

### Statistical analysis

Data were analyzed using the Student’s t-test, Mann–Whitney U test, Wilcoxon’s signed-rank test, the χ2 test, Fisher’s exact test, or analysis of variance, as appropriate. Time-to-mortality and time-to-acute exacerbation were evaluated with Kaplan–Meier survival curves. Log-rank test was performed to compare the groups. Additionally, multivariate Cox proportional-hazard regression analyses were implemented. Variables with *P* < 0.20 in univariate analysis were selected for multivariate analysis.

A value of *P* < 0.05 was considered statistically significant (two-tailed). All statistical analyses were conducted using R Statistical Software (version 4.0.3; R Foundation for Statistical Computing, Vienna, Austria).

## Results

### Baseline characteristics

**Fig. 1:**
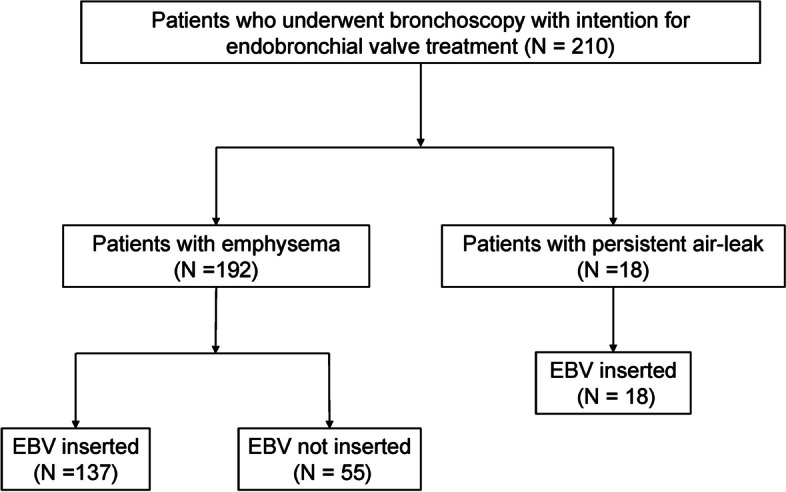
Flow chart of the study patients selection process. Abbreviation: EBV, endobronchial valve

A total of 210 patients were included in the study (Supplementary Fig. 1, Additional file 2). The majority (91.4%) had severe emphysema, and the rest (8.6%) had PAL (Fig. 1). The mean age of the entire population, including 95.7% male patients, was 69.7 years. The median follow-up period was 18.5 months for patients with emphysema and 8.0 months for those with PAL (Table 1).

**Table 1:** Baseline characteristics of the study population

Characteristics	Number
Total number of patients	210
Age, years	69.7 ± 10.8
Male sex	201 (95.7)
Body mass index, kg/m^2^	19.8 ± 3.6
Smoking status	
Ex-smoker	175 (83.3)
Current smoker	22 (10.5)
Never smoker	12 (5.7)
Unknown	1 (0.5)
Past medical history	
Bronchiectasis	3 (1.4)
Asthma	15 (7.1)
Ischemic heart disease	5 (2.4)
Heart failure	5 (2.4)
Hypertension	52 (24.8)
Diabetes mellitus	30 (14.3)
Stroke	5 (2.4)
Long-term oxygen therapy	77 (36.7)
Indications for EBV	
Lung volume reduction	192 (91.4)
Persistent air-leak	18 (8.6)

Data are presented as mean ± standard deviation, median (interquartile range) or number (%)

*Abbreviation: EBV* Endobronchial valve

Among the 192 patients with emphysema, EBV was placed in 137 (71.4%). Distribution of age (72.6 years vs. 69.4 years, *P* = 0.053), sex (male: 96.4% vs. 95.6%, *P* > 0.999), and residual volume (209.3% vs. 197.6% predicted, *P* = 0.247) were comparable between the no-BLVR and BLVR groups; however, BMI was lower in the no-BLVR group (18.7 kg/m^2^ vs. 20.1 kg/m^2^, *P* = 0.013). Additionally, FEV_1_ after bronchodilation (25.2% predicted vs. 31.4% predicted, *P* = 0.001) and the diffusing capacity of the lungs for carbon monoxide (5.6 min∙mm Hg vs. 7.1 min∙mm Hg, *P* = 0.002) were lower in the no-BLVR group than in the BLVR group. Baseline comorbidities, long-term oxygen therapy, use of inhalers, COPD assessment test scores and mMRC dyspnea scale scores were similar (Supplementary Table 1, Additional file 3).

In the 18 (94.4% male) patients with PAL, the mean age was 63.4 years and mean BMI was 21.6 kg/m^2^ (Table 2). Fifteen patients (83.3%) had been previously diagnosed with chest diseases, and the most common cause of PAL was spontaneous secondary pneumothorax (8/18, 44.4%), followed by parapneumonic effusion/empyema (4/18, 22.2%) and surgical resection of the lung (3/18, 16.7%) (Supplementary Table 3, Additional file 5). Within the cohort, 17 patients retained chest tubes during the procedure. The sole patient without a chest tube at the time of the procedure exhibited an air leak at the pneumonectomy site. Four patients had previously undergone surgical intervention for PAL, which did not result in the resolution of the condition.

**Table 2:** Characteristics and outcomes of patients with persistent air-leak who underwent endobronchial valve treatment (*N* = 18)

Characteristics	Numbers
Age, years	63.4 ± 13.1
Male sex	17 (94.4)
Body mass index, kg/m^2^	21.6 ± 4.2
Causes of air-leak	
Spontaneous secondary pneumothorax	8 (44.4)
Parapneumonic effusion/empyema	4 (22.2)
Lung resection surgery	3 (16.7)
Bronchobiliary fistula	1 (5.6)
Mechanical ventilation, underlying emphysema	1 (5.6)
Unknown	1 (5.6)
Adverse events	
Hemoptysis	2 (11.1)
Non-massive	1 (5.6)
Massive	1 (5.6)
Empyema	1 (5.6)
Outcomes	
Improvement in air-leak	14 (77.8)
Time to resolution of pneumothorax on chest radiograph, days	3 (0–19.5)
Time to EBV after chest tube insertion, days	25 (10.0–49.5)
Time to chest tube removal after EBV insertion, days	16.5 (4.75–33.5)
Total time of chest tube retention, days	46.5 (23.3–79.0)

Data are presented as mean ± standard deviation or number (%) or median (interquartile range) of patients

*Abbreviation*: *EBV* Endobronchial valve

### Procedure details

EBV insertion was performed on 155 patients. The right upper lobe was the most frequent location for placement. Most procedures (74.2%) were performed under conscious sedation. The emphysema group exhibited increased number of valves placement than the PAL group (3.0 vs. 2.0, *P* < 0.001). The mean duration of the procedure was 44.3 min, and it was comparable between the patients with emphysema and PAL (45.1 vs. 38.0 min, *P* = 0.346). The mean hospital stay after the procedure was 6.1 and 30.3 days, respectively (Supplementary Table 2. Additional file 4).

### Adverse events

Pneumothorax (14.1%) was the most common adverse event in 192 patients with severe emphysema. It was more frequent in the BLVR group (18.9% vs. 3.8%, *P* = 0.018). In the BLVR group, 52.0% (13/25) had pneumothorax within 7 days of the procedure (median time: 1 day, interquartile range [IQR]: 1–2 days), whereas 48.0% (12/25) had more delayed pneumothorax (median time: 108 days, IQR: 36.8–245.5 days). EBVs were removed in six patients, and one patient died of tension pneumothorax. The occurrence of hemoptysis (1.9% vs. 9.2%, *P* = 0.165) and empyema did not differ between the groups (1.9% vs. 0.8%, *P* > 0.999, Table 3).

**Table 3:** Adverse event in patients who underwent bronchoscopy with intention for bronchoscopic lung volume reduction

Characteristics	Total	No-BLVR	BLVR	*P*
Number of patients	192	55	137	
Pneumothorax	27 (14.1)	2 (3.8)	25 (18.9)	0.018
Hemoptysis	13 (6.8)	1 (1.9)	12 (9.1)	0.165
Empyema	2 (1.0)	1 (1.9)	1 (0.8)	> 0.999
Acute exacerbation of COPD	90 (46.9)	20 (36.4)	70 (51.1)	0.091
Mortality	69 (35.9)	30 (54.5)	39 (28.5)	0.001
Procedure-related	1 (0.5)	0 (0.0)	1 (0.7)	
Not procedure-related	68 (99.5)	30 (100.0)	136 (99.3)	

Data are presented as number (%)

*Abbreviations*: *BLVR* Bronchoscopic lung volume reduction, *COPD* Chronic obstructive pulmonary disease

In the cohort of patients with PAL, post-procedural complications included hemoptysis in two cases (11%), including one case of non-massive and one of massive hemoptysis. Additionally, one patient (5.6%) developed empyema. No further adverse events were noted (Table 2).

### Clinical outcomes of COPD emphysema

**Fig. 2:**
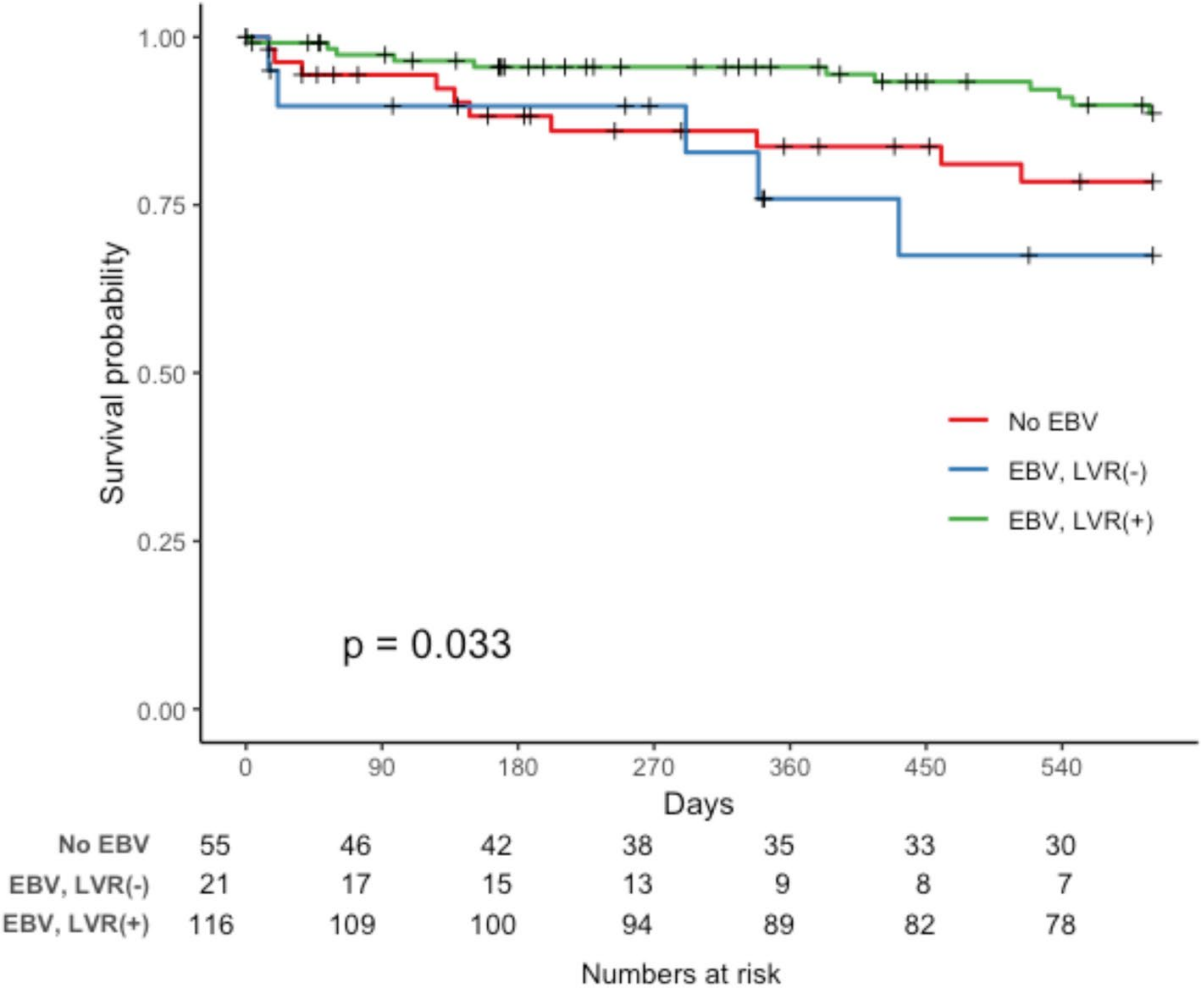
Kaplan–Meier survival curves for patients with severe emphysema. Abbreviation: BVLR, bronchoscopic lung volume reduction

For patients with severe emphysema, overall survival rates were comparable between those who underwent BLVR and those who did not (*P* = 0.181, Supplementary Fig. 2, Additional file 6). However, a subgroup analysis revealed a significant difference in survival between the no-BLVR and BLVR groups categorized based on radiographic evidence of lung volume reduction 6 months after the procedure (*P* = 0.033, Fig. 2). Kaplan–Meier survival curve analysis indicated similar survival trends between patients in the no-BLVR group and those in the BLVR group without lung volume reduction. Radiographic lung volume reduction at 6 months was an independent prognostic factor for reduced mortality after adjusting for age, sex, BMI, baseline FEV1 (% predicted), and mMRC dyspnea scale scores (adjusted hazard ratio [aHR], 0.020; 95% CI 0.038–0.650; *P* = 0.010, Table 4). The incidence of COPD acute exacerbation was not significantly different between the BLVR group and no-BLVR group (*P* = 0.120, Supplementary Fig. 3, Additional file 7).

**Table 4:** Cox proportional hazard regression analysis for mortality

	Univariate analysis			Multivariate analysis		
	HR	95% CI	*P*	aHR	95% CI	*P*
Age, years	1.022	0.974–1.073	0.378			
Female sex	3.194	0.725–14.06	0.125	1.572	0.295–8.381	0.597
Body mass index	0.797	0.679–0.935	0.005	0.749	0.595–0.944	0.014
FEV_1_, % predicted	0.201	0.030–1.365	0.101	0.523	0.092–2.989	0.466
MMRC grade	1.291	0.612–2.722	0.503			
Radiographic reduction in lung volume by visual estimation	0.282	0.010–0.816	0.020	0.156	0.038–0.650	0.010

*Abbreviations*: *FEV*_*1*_ Forced expiratory volume in 1 s, *MMRC* Modified Medical Research Council, *HR* Hazard ratio, *CI* Confidence interval, *aHR* Adjusted hazard ratio

**Fig. 3:**
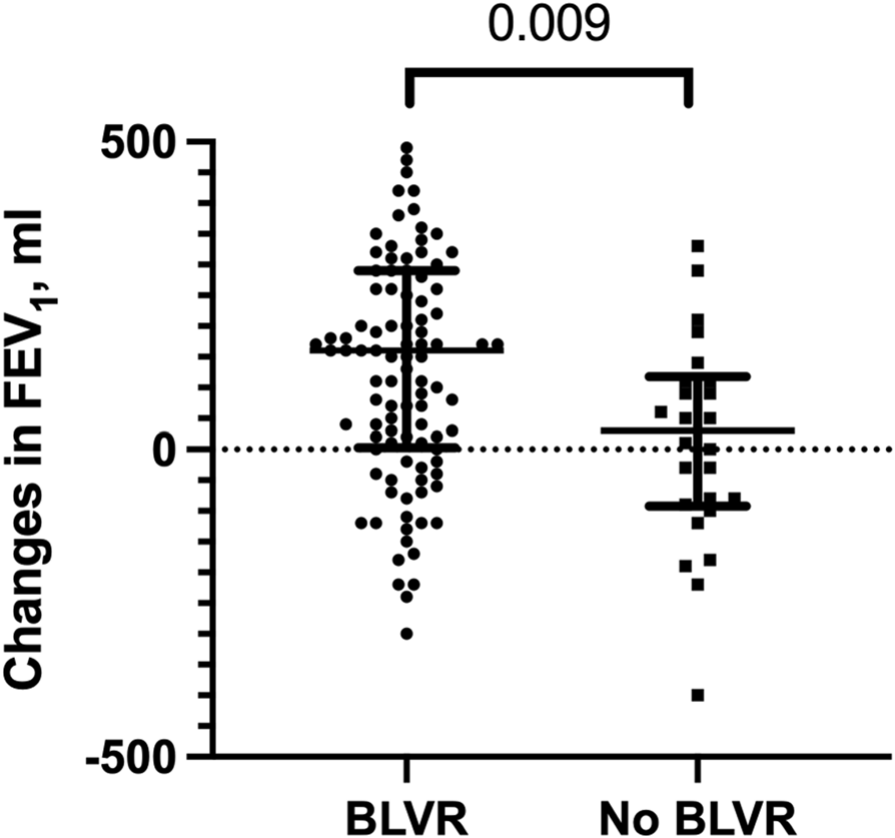
Changes in FEV_1_ 3 months after evaluation for BLVR. Abbreviations: FEV_1_, forced expiratory volume in 1 s, BVLR, bronchoscopic lung volume reduction

Three months post-procedure, the FEV_1_ improvement was more pronounced in the BLVR groups than in the no-BLVR group (+160 mL vs. +30 mL; *P* = 0.009, Fig. 3). Within the BLVR group, a 20.0% enhancement of FEV_1_ was noted. On day 300, the FEV1 continued to rise in the BLVR group, whereas it declined in the no-BLVR group (Supplementary Fig. 4, Additional file 8).

### Clinical outcomes of persistent air-leak

All the 18 patients underwent CT scans to determine the appropriate sites for EBV insertion. In ten of these patients, indigo carmine blue dye was utilized to further delineate the air-leak sites. Collateral ventilation assessment using the Chartis system was conducted in four patients, which did not reveal any collateral ventilation. Among the 17 patients who had a chest tube at the time of the procedure, 14 (82.4%) had their chest tubes removed after treatment. The median interval from the procedure, as evidenced on chest radiographs, was 3.0 days (IQR: 0–19.5 days). The median time to chest tube removal following the procedure was 16.5 days (IQR: 4.75–33.50). Among the four patients evaluated using the Chartis system, chest tube removal occurred on 2, 4, and 40 days in three patients, respectively, after EBV insertion. The chest tube could not be removed in another patient. Three patients (17.6%) in total were unable to have their chest tubes removed. The causes of the air-leak were spontaneous secondary pneumothorax and bronchobiliary fistula, and it was unknown in one patient.

The valves were removed in two cases. In one case, the valves were removed after the resolution of PAL. In the other case, they were removed after treatment with EBV was considered ineffective. No adverse events related to the procedure were noted in both cases. Additionally, one patient reported expelling the valve during a coughing episode at a follow-up outpatient clinic visit.

## Discussion

In this analysis, we reviewed the cases of 210 patients assessed for EBV therapy, of which 155 underwent the procedure. Specifically, BLVR was performed in 137 out of 192 patients with severe emphysema who were evaluated as potential candidates. Notably, an independent predictor of improved overall survival was the radiographic evidence of lung volume reduction, as determined by visual estimation. Furthermore, in 18 patients with PAL, a majority (77.8%) exhibited clinical improvement following treatment with EBV.

Among patients with emphysema, we observed a larger increase in FEV_1_ and a trend toward better survival in patients who underwent BLVR than in those who did not. The observed 20% improvement in FEV_1_ after 3 months is consistent with previous studies, which have reported improvements ranging from 17 to 29% [13]. Although we were unable to evaluate long-term changes in FEV_1_ due to insufficient follow-up PFT data, the trend was sustained up to approximately 1 year. The multicenter randomized controlled LIBERATE trial showed an FEV_1_ increase of 104 mL after a year of the treatment with EBV [3]. Another recent study including 280 patients reported that the improvements in FEV_1_ and other clinical outcome measures were maintained at least up to 3 years, albeit with a diminishing effect over time [14].

Improved survival with BLVR has been consistently reported in the literature. In the subgroup analysis in the STELVIO study, Klooster et al. found that predictors of survival, the BODE index score, 6-min-walk distance, and hyperinflation improve after BLVR [15]. In another retrospective cohort study comparing 483 patients in the BLVR group and 988 patients in the no-BLVR group, the median survival was longer in the BLVR group (median 3133 days vs. 2503 days; *P* < 0.001) [16].

BLVR treatment is usually performed in carefully selected patients with severe emphysema who remain symptomatic despite optimal medical treatment, show evidence of hyperinflation, and demonstrated absence of CV [13, 17]. However, even among the carefully selected patients, treatment response is variable. In our subgroup analysis, we found radiographic reduction in lung volume assessed through visual estimation at 6 months interval to be an independent factor associated with better survival. Nonetheless, survival benefit was not observed without lung volume reduction (Fig. 2). Based on the result, we re-emphasize the importance of evaluating BLVR candidates for their potential to achieve lung volume reduction.

Studies on the off-label use of Zephyr EBV in patients with PAL is limited. However, most of the patients (82.4%) included in this study for PAL were able to have their chest tube removed after placement of EBVs. Similarly, an Italian multicenter retrospective study including 67 patients who had PAL after lung resection, reported a resolution rate of 88.0% after the treatment [18]. Although the treatment success rate was lower in our cohort compared with that is previous studies, this was anticipated since we included all causes of PAL rather than limiting to post-surgical cases. Currently, the FDA has only approved the use of the intrabronchial valve (Spiration. Inc., Redmond, Washington, USA) for managing post-surgical prolonged air-leaks under the Human Device Exemption (HDE) program [19]. Nevertheless, attempts to treat PAL of various causes with bronchoscopies continue, as PAL is detrimental, and its management is still controversial and challenging [20–23]. Our study adds to the feasibility of managing PAL of different causes with EBV. However, further prospective studies with controlled protocols are warranted, especially for assessing treatment response in the presence of collateral ventilation.

Reports on BLVR from Asian countries, including Korea, Japan, and China, are limited. In Japan, BLVR techniques with autologous blood, thrombin or Spigot have been reported through case series or reports [24, 25], and relatively common methods such as EBV or coil have not been published. In China, experimental devices including flap or occlude have been studied [26, 27], alongside case reports [28, 29] and single-arm studies evaluating the efficacy of endobronchial valve [30, 31], showing improvements in lung function and exercise capacity. In Korea, after the early studies reporting the clinical outcomes of EBV insertion [9, 10], studies on the improvement of ventilation-perfusion mismatch with BLVR and the utility of fissure integrity analyzed through quantitative CT to select EBV insertion sites were published [32, 33]. Essentially, the present evidence of BLVR is mostly based on data from the non-Asian population. In this context, our study showed meaningful insights into BLVR, drawn from data obtained from more than two hundred patients with a median follow-up of 18.5 months, confirming the long-term efficacy and safety of this treatment in the Asian population.

In the general population, lung function differs among racial groups. Caucasians have larger total lung capacity, forced vital capacity, and FEV_1_, whereas functional residual capacity and residual volume are similar [34, 35]. Although the cause of the disparity is inconclusive, the differences have been attributed to anatomical distinctions. In a study comparing 13 Caucasians, 14 Chinese, and 11 Indian, the width and surface area of the Caucasian were found to be greater [36]. Our study has shown BLVR can be implemented in Asian patients, despite the distinctions.

This study has few limitations. First, this is a retrospective review, limiting the evaluation of confirmative effectiveness. However, the inclusion of data from more than 90% of patients who underwent treatment in one countryprovides valuable insights into how this procedure was conducted and standardized after being introduced to the country. Second, the procedure was performed in 11 institutions, and the protocols were not standardized. For instance, while most institutions performed BLVR under local anesthesia after confirming the absence of CV using the Chartis system, one institution performed BLVR mostly under general anesthesia (35/41, 85.4%) without Chartis evaluation (28/41, 68.3%). Furthermore, the use of the Chartis system for patients with PAL varied as three of the six institutes used it and three did not. These differences can contribute in part to the results that survival benefit was only evident when the target lobar volume was reduced, and EBV insertion alone did not result in sufficient differences. Third, initially, we planned to incorporate the analysis of mMRC dyspnea scale and 6-min-walk distance test to assess functional improvements in patients following BLVR. However, due to the gaps in data, we were unable to evaluate serial changes in those outcomes. Instead, we focused on a comprehensive evaluation of PFT.

## Conclusions

We reviewed data from 210 patients considered for treatment with EBV in an Asian country over 10 years, with 155 eventually receiving the valve. Most were patients with severe emphysema. FEV_1_ improvements were significantly greater in the BLVR group than in the no-BLVR group. Furthermore, a trend for better survival was observed in the BLVR group than in the no-BLVR group. For patients with PAL, treatment with EBV showed satisfactory resolution rate with few adverse events. This study suggests that treatment with EBV is an effective and safe option in the Asian population.

### Supplementary Information

**Additional file 1.** Institutional review board protocol numbers. Documentation of the IRB protocol numbers.

**Additional file 2: Supplementary Figure 1.** Number of cases from each hospital included in this study.

**Additional file 3: Supplementary Table 1.** Baseline characteristics of patients who underwent bronchoscopy for intended bronchoscopic lung volume reduction.

**Additional file 4: Supplementary Table 2.** Procedural details of patients who received treatment with EBV.

**Additional file 5: Supplementary Table 3.** Comorbidities of patients who underwent treatment with EBV for persistent air-leak.

**Additional file 6: Supplementary Figure 2.** Kaplan–Meier survival analysis of the overall survival among patients with severe emphysema following treatment with EBV.

**Additional file 7: Supplementary Figure 3.** Kaplan–Meier survival curves for COPD acute exacerbation in patients with severe emphysema who underwent treatment with EBV.

**Additional file 8: Supplementary Figure 4.** Changes in FEV_1_ over 300 days in patients with severe emphysema.

## Data Availability

The datasets used and/or analyzed during the current study are available from the corresponding author on reasonable request.

## Abbreviations

EBV: Endobronchial valve
BLVR: Bronchoscopic lung volume reduction
PAL: Persistent air-leak
CV: Collateral ventilation
BMI: Body mass index
FEV_1_: Forced expiratory volume in 1 s
CT: Computed tomography
IRB: Institutional review board
PFT: Pulmonary function test
COPD: Chronic obstructive pulmonary disease
mMRC Dyspnea Scale: Modified Medical Research Council Dyspnea Scale
IQR: Interquartile range
