# Supplementary material for: Endobronchial valves for emphysema and persistent air-leak: 10-year experience in an Asian country
Source: BMC Pulm Med. 2024 Apr 3;24:162. doi: 10.1186/s12890-024-02982-2 (PMC10988911; doi:10.1186/s12890-024-02982-2)
Supplement: Supplementary file 1 — Additional file 1. Institutional review board protocol numbers. Documentation of the IRB protocol numbers. [file 12890_2024_2982_MOESM1_ESM.docx]

**INSTITUTIONAL REVIEW BOARD PROTOCOL NUMBERS**

Asan Medical Center: 2021-0317

Samsung Medical Center: 2021-01-087-001

Seoul National University Bundang Hospital: B-2112-729-402

Jeju National University Hospital: 2021-08-005

Gyeongsang National University Hospital: 2021-12-021

Eunpyeong St. Mary’s Hospital: PC21RIDI0125

Ulsan University Hospital: UUH 2021-10-020

Seoul National University Hospital: J-2301-129-1398

Korea University Ansan Hospital: 2021AS0308

St. Vincent Hospital: VC23RIDI0021

Soonchunhyang University Cheonan Hospital: 2021-08-030
