# Supplementary material for: Endobronchial valves for emphysema and persistent air-leak: 10-year experience in an Asian country
Source: BMC Pulm Med. 2024 Apr 3;24:162. doi: 10.1186/s12890-024-02982-2 (PMC10988911; doi:10.1186/s12890-024-02982-2)
Supplement: Supplementary file 2 — Additional file 2: Supplementary Figure 1. Number of cases from each hospital included in this study. [file 12890_2024_2982_MOESM2_ESM.docx]

Supplementary Figure 1. Number of cases from each hospital included in this study


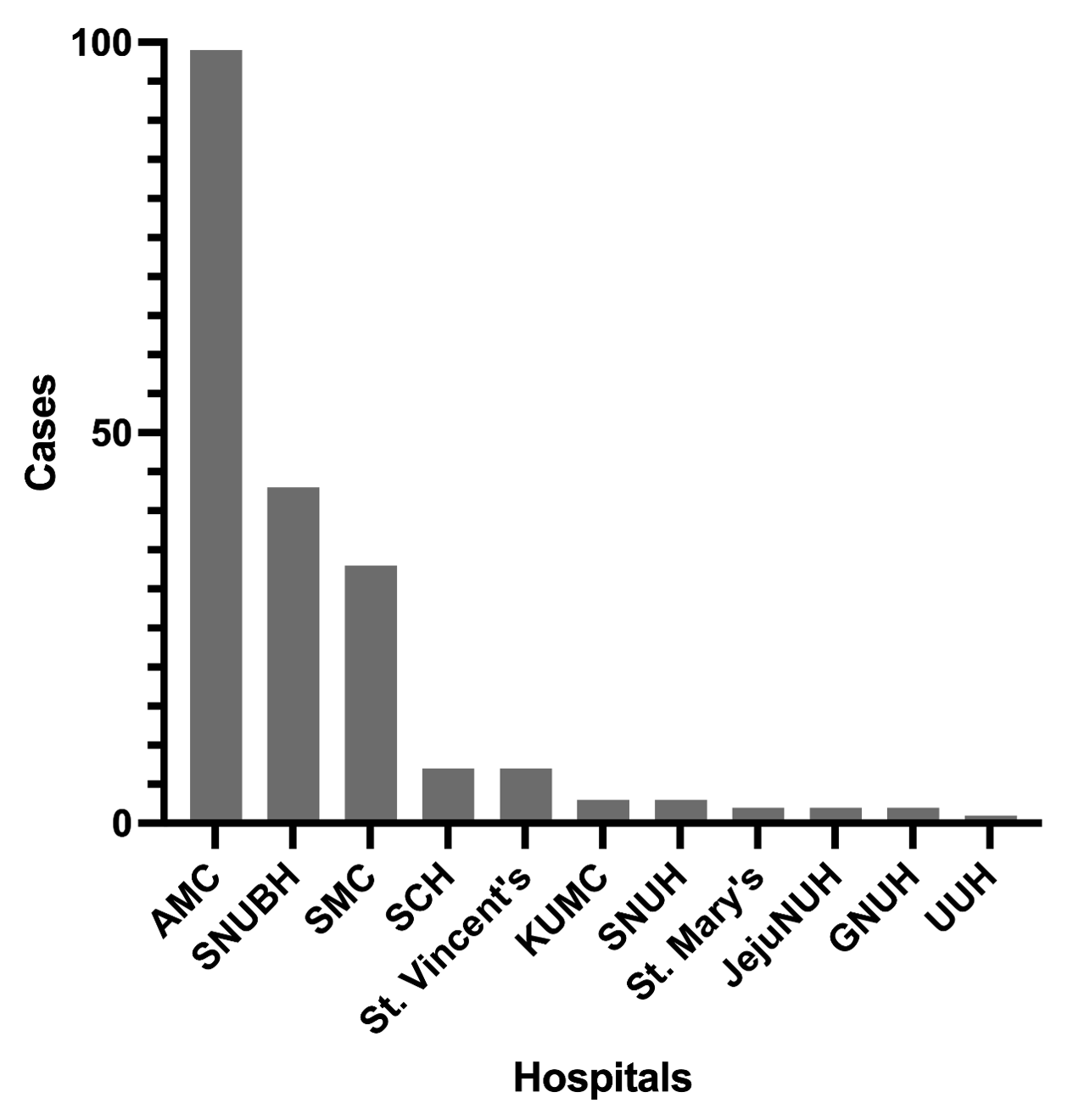


*Abbreviations*: AMC, Asan Medical Center; SNUBH, Seoul National University Bundang Hospital; SMC, Samsung Medical Center; SCH, Soonchunhyang University Cheonan Hospital; KUMC, Korea University Ansan Hospital; SNUH, Seoul National University Hospital; St Mary’s, Eunpyeong St. Mary’s Hospital; Jeju NUH, Jeju National University Hospital; GNUH, Gyeongsang National University Hospital; UUH, Ulsan University Hospital
