# Supplementary material for: Endobronchial valves for emphysema and persistent air-leak: 10-year experience in an Asian country
Source: BMC Pulm Med. 2024 Apr 3;24:162. doi: 10.1186/s12890-024-02982-2 (PMC10988911; doi:10.1186/s12890-024-02982-2)
Supplement: Supplementary file 3 — Additional file 3: Supplementary Table 1. Baseline characteristics of patients who underwent bronchoscopy for intended bronchoscopic lung volume reduction. [file 12890_2024_2982_MOESM3_ESM.docx]

Supplementary Table 1. Baseline characteristics of patients who underwent bronchoscopy for intended bronchoscopic lung volume reduction

| Characteristics | Total | No-BLVR | BLVR | *P* |
| --- | --- | --- | --- | --- |
| Number of patients | 192 | 55 | 137 |  |
| Age, years | 70.3 ± 10.4 | 72.6 ± 9.1 | 69.4 ± 10.8 | 0.053 |
| Male sex | 184 (95.8) | 53 (96.4) | 131 (95.6) | >0.999 |
| Body mass index, kg/m^2^ | 19.7 ± 3.5 | 18.7 ± 3.2 | 20.1 ± 3.5 | 0.013 |
| Smoking status |  |  |  | 0.442 |
| Ex-smoker | 166 (86.4) | 49 (89.1) | 117 (85.4) |  |
| Current smoker | 18 (9.4) | 3 (5.5) | 15 (10.9) |  |
| Never smoker | 8 (4.2) | 3 (5.5) | 5 (3.6) |  |
| Smoking amount, pack-years | 46.0 ± 22.5 | 48.0 ± 19.8 | 45.2 ± 23.5 | 0.457 |
| Past medical history |  |  |  |  |
| Oxygen therapy | 75 (39.1) | 25 (45.5) | 50 (36.5) | 0.324 |
| Hypertension | 47 (24.5) | 14 (25.5) | 33 (24.1) | 0.989 |
| Diabetes mellitus | 26 (13.5) | 7 (12.7) | 19 (13.9) | >0.999 |
| Asthma | 15 (7.8) | 4 (7.3) | 11 (8.0) | >0.999 |
| Ischemic heart disease | 5 (2.6) | 1 (1.8) | 2 (1.5) | >0.999 |
| Stroke | 5 (2.6) | 1 (1.8) | 4 (2.9) | >0.999 |
| Heart failure | 3 (1.6) | 1 (1.8) | 2 (1.5) | >0.999 |
| Bronchiectasis | 2 (1.0) | 1 (1.8) | 1 (0.7) | >0.999 |
| Pulmonary function test |  |  |  |  |
| Before bronchodilator |  |  |  |  |
| FEV_1_, liter | 0.8 ± 0.5 | 0.7 ± 0.3 | 0.9 ± 0.5 | <0.001 |
| FEV_1_, % predicted | 28.6 ± 14.6 | 23.7 ± 8.4 | 30.5 ± 16.1 | <0.001 |
| FVC, liter | 2.6 ± 0.8 | 2.4 ± 0.7 | 2.6 ± 0.8 | 0.118 |
| FVC, % predicted | 64.9 ± 17.8 | 62.1 ± 17.8 | 66.0 ± 17.7 | 0.178 |
| After bronchodilator |  |  |  |  |
| FEV_1_, liter | 0.8 ± 0.4 | 0.7 ± 0.3 | 0.9 ± 0.5 | 0.002 |
| FEV_1_, % predicted | 29.6 ± 13.3 | 25.2 ± 9.4 | 31.4 ± 14.2 | 0.001 |
| FVC, liter | 3.2 ± 7.7 | 4.6 ± 14.5 | 2.7 ± 0.8 | 0.375 |
| FVC, % predicted | 66.7 ± 17.8 | 62.7 ± 20.6 | 68.3 ± 16.4 | 0.089 |
| RV, liter | 5.0 ± 4.2 | 5.9 ± 7.4 | 4.5 ± 1.2 | 0.214 |
| RV, % predicted | 201.2 ± 55.0 | 209.3 ± 63.9 | 197.6 ± 50.5 | 0.247 |
| TLC, liter | 7.1 ± 1.3 | 7.2 ± 1.2 | 7.1 ± 1.3 | 0.501 |
| TLC, % predicted | 123.5 ± 20.5 | 124.2 ± 18.5 | 123.1 ± 21.6 | 0.776 |
| DL_CO_, mL/min∙mm Hg | 6.7 ± 3.2 | 5.6 ± 2.3 | 7.1 ± 3.5 | 0.002 |
| DL_CO_, % predicted | 38.2 ± 35.2 | 31.8 ± 15.7 | 40.9 ± 40.7 | 0.045 |
| Six-minute walk distance | 243.8 ± 123.4 | 226.3 ± 113.6 | 253.3 ± 128.1 | 0.260 |
| COPD assessment test score | 27.1 ± 6.9 | 27.8 ± 6.2 | 27.0 ± 7.0 | 0.720 |
| MMRC dyspnea scale |  |  |  | 0.111 |
| Grade 1 | 4 (2.7) | 0 (0.0) | 4 (3.9) |  |
| Grade 2 | 20 (13.7) | 4 (9.3) | 16 (15.5) |  |
| Grade 3 | 48 (32.9) | 11 (25.6) | 37 (35.9) |  |
| Grade 4 | 74 (50.7) | 28 (65.1) | 46 (44.7) |  |
| Use of inhalers, total | 177 (92.2) | 52 (94.5) | 125 (91.2) | 0.636 |
| Inhaler classes |  |  |  | 0.893 |
| ICS/LABA/LAMA | 103 (58.2) | 28 (53.8) | 75 (60.0) |  |
| LAMA/LABA | 30 (16.9) | 10 (19.2) | 20 (16.0) |  |
| LAMA | 21 (11.9) | 6 (11.5) | 15 (12.0) |  |
| ICS/LABA | 18 (10.2) | 7 (13.5) | 11 (8.8) |  |
| SABA, as needed | 2 (1.1) | 1 (1.9) | 1 (0.8) |  |
| Others | 3 (1.7) | 0 (0.0) | 3 (2.4) |  |

Data are presented as mean ± standard deviation, median (interquartile range) or number (%).

*Abbreviations:* BLVR, bronchoscopic lung volume reduction; FEV_1_, forced expiratory volume in 1 second; FVC, forced vital capacity; RV, residual volume; TLC, total lung capacity; DL_CO_, diffusing capacity of the lungs for carbon monoxide; COPD, chronic obstructive pulmonary disease; MMRC, modified Medical Research Council; ICS, inhaled corticosteroid; LAMA, long-acting muscarinic antagonist; LABA, long-acting β-agonist; SABA, short-acting β-agonist; ICS, inhaled corticosteroid.

^*^Number of cigarettes smoked among ever-smokers.
