# Supplementary material for: Endobronchial valves for emphysema and persistent air-leak: 10-year experience in an Asian country
Source: BMC Pulm Med. 2024 Apr 3;24:162. doi: 10.1186/s12890-024-02982-2 (PMC10988911; doi:10.1186/s12890-024-02982-2)
Supplement: Supplementary file 4 — Additional file 4: Supplementary Table 2. Procedural details of patients who received treatment with EBV. [file 12890_2024_2982_MOESM4_ESM.docx]

Supplementary Table 2. Procedural details of patients who received treatment with EBV

| Characteristics | Total | Emphysema | Persistent air-leak | *P* |
| --- | --- | --- | --- | --- |
| Number of patients | 155 | 137 | 18 |  |
| Location of EBV |  |  |  |  |
| RUL | 59 (38.1) | 51 (37.2) | 8 (44.4) | 0.738 |
| RBI | 3 (1.9) | 2 (1.5) | 1 (5.6) | 0.783 |
| RML | 7 (4.5) | 6 (4.4) | 1 (5.6) | >0.99 |
| RLL | 28 (18.1) | 26 (19.0) | 2 (11.1) | 0.624 |
| LUL | 31 (20.0) | 27 (19.7) | 4 (22.2) | >0.99 |
| LLL | 37 (23.9) | 36 (26.3) | 1 (5.6) | 0.100 |
| Number of inserted valves | 2.9 ± 1.1 | 3.0 ± 1.1 | 2.0 ± 0.7 | <0.001 |
| Anesthesia |  |  |  | 0.102 |
| Conscious sedation | 115 (74.2) | 105 (76.6) | 10 (55.6) |  |
| General anesthesia | 40 (25.8) | 32 (23.4) | 8 (44.4) |  |
| Procedure duration (minutes) | 44.3 ± 28.3 | 45.1 ± 28.2 | 38.0 ± 29.2 | 0.346 |
| In-hospital days after procedure | 8.8 ± 22.2 | 6.1 ± 7.8 | 30.3 ± 59.2 | 0.111 |

Data are presented as mean ± standard deviation.

*Abbreviations*: EBV, endobronchial valve; RUL, right upper lobe; RBI, right bronchus intermedius; RML, right middle lobe; RLL, right lower lobe; LUL, left upper lobe; LLL, left lower lobe
