# Supplementary material for: Endobronchial valves for emphysema and persistent air-leak: 10-year experience in an Asian country
Source: BMC Pulm Med. 2024 Apr 3;24:162. doi: 10.1186/s12890-024-02982-2 (PMC10988911; doi:10.1186/s12890-024-02982-2)
Supplement: Supplementary file 5 — Additional file 5: Supplementary Table 3. Comorbidities of patients who underwent treatment with EBV for persistent air-leak. [file 12890_2024_2982_MOESM5_ESM.docx]

Supplementary Table 3. Comorbidities of patients who underwent treatment with EBV for persistent air-leak

| Patient | Age, years/Sex | Past medical history | Bulla | History of other chest disease | Cause of air-leak |
| --- | --- | --- | --- | --- | --- |
| 1 | 65/Male | Diabetes mellitus | + | - | Spontaneous secondary pneumothorax |
| 2 | 64/Male |  | + | Emphysema | Mechanical ventilation |
| 3 | 58/Male |  | + | Pneumothorax | Spontaneous secondary pneumothorax |
| 4 | 53/Male |  | + | Emphysema | Spontaneous secondary pneumothorax |
| 5 | 66/Male |  | - | Emphysema, NTM pulmonary disease | Spontaneous secondary pneumothorax |
| 6 | 72/Male | Hypertension | + | Pneumothorax, Emphysema | Spontaneous secondary pneumothorax |
| 7 | 76/Male | Heart failure, diabetes mellitus | + | Lung cancer, Emphysema | Spontaneous secondary pneumothorax |
| 8 | 63/Male |  | - | Necrotizing pneumonia due to *Klebsiella pneumoniae* | Parapneumonic/empyema |
| 9 | 67/Male |  | - | Lung cancer | Postoperative |
| 10 | 72/Male |  | + | Emphysema, Pneumothorax | Spontaneous secondary pneumothorax |
| 11 | 21/Male | Marfan syndrome | - | Pectus excavatum | Parapneumonic effusion/empyema |
| 12 | 56/Female |  | - | Mesothelioma | Postoperative |
| 13 | 65/Male | Hypertension | - | - | Parapneumonic effusion/empyema |
| 14 | 54/Male |  | - | Colon cancer lung metastasis | Postoperative |
| 15 | 70/Male | Diabetes mellitus | - | - | Bronchobiliary fistula |
| 16 | 75/Male | Hypertension, diabetes mellitus | + | Pneumothorax, Lung cancer | Unknown |
| 17 | 82/Male | Hypertension | - | Empyema | Parapneumonic effusion/empyema |
| 18 | 62/Male | Hypertension | - | Lung cancer, COPD | Spontaneous secondary pneumothorax |

*Abbreviations*: EBV, endobronchial valve; NTM, non-tuberculous mycobacteria, COPD, chronic obstructive pulmonary disease
