# Supplementary material for: Endobronchial valves for emphysema and persistent air-leak: 10-year experience in an Asian country
Source: BMC Pulm Med. 2024 Apr 3;24:162. doi: 10.1186/s12890-024-02982-2 (PMC10988911; doi:10.1186/s12890-024-02982-2)
Supplement: Supplementary file 6 — Additional file 6: Supplementary Figure 2. Kaplan–Meier survival analysis of the overall survival among patients with severe emphysema following treatment with EBV. [file 12890_2024_2982_MOESM6_ESM.docx]

Supplementary Figure 2. Kaplan–Meier survival analysis of the overall survival among patients with severe emphysema following treatment with EBV


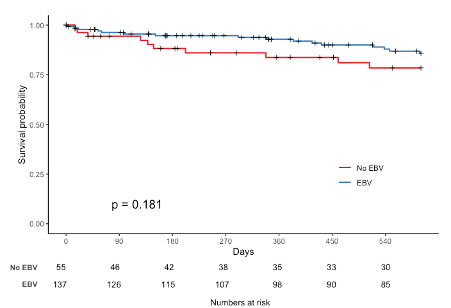


*Abbreviations*: EBV, endobronchial valve
