# Supplementary material for: Endobronchial valves for emphysema and persistent air-leak: 10-year experience in an Asian country
Source: BMC Pulm Med. 2024 Apr 3;24:162. doi: 10.1186/s12890-024-02982-2 (PMC10988911; doi:10.1186/s12890-024-02982-2)
Supplement: Supplementary file 7 — Additional file 7: Supplementary Figure 3. Kaplan–Meier survival curves for COPD acute exacerbation in patients with severe emphysema who underwent treatment with EBV. [file 12890_2024_2982_MOESM7_ESM.docx]

Supplementary Figure 3. Kaplan–Meier survival curves for COPD acute exacerbation in patients with severe emphysema who underwent treatment with EBV


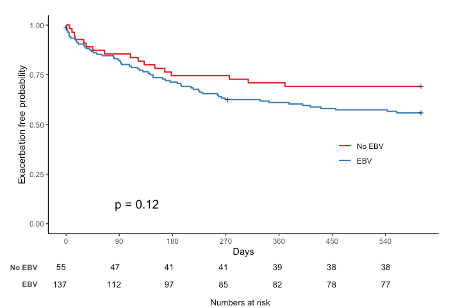


*Abbreviations*: COPD, chronic obstructive pulmonary disease; EBV, endobronchial valve
