# Supplementary material for: Endobronchial valves for emphysema and persistent air-leak: 10-year experience in an Asian country
Source: BMC Pulm Med. 2024 Apr 3;24:162. doi: 10.1186/s12890-024-02982-2 (PMC10988911; doi:10.1186/s12890-024-02982-2)
Supplement: Supplementary file 8 — Additional file 8: Supplementary Figure 4. Changes in FEV1 over 300 days in patients with severe emphysema. [file 12890_2024_2982_MOESM8_ESM.docx]

Supplementary Figure 4. Changes in FEV_1_ over 300 days among patients with severe emphysema


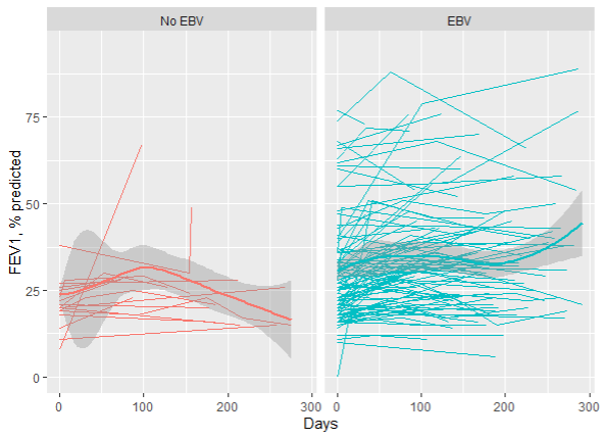


*Abbreviations*: FEV_1_, forced expiratory volume in 1 second; EBV, endobronchial valve
